# Sauti ya Vijana (SYV; The Voice of Youth): Longitudinal Outcomes of an Individually Randomized Group Treatment Pilot Trial for Young People Living with HIV in Tanzania

**DOI:** 10.1007/s10461-021-03550-z

**Published:** 2022-01-24

**Authors:** Dorothy E. Dow, Karen E. O’Donnell, Laura Mkumba, John A. Gallis, Elizabeth L. Turner, Judith Boshe, Aisa M. Shayo, Coleen K. Cunningham, Blandina T. Mmbaga

**Affiliations:** 1 Department of Pediatrics, Infectious Diseases, Duke University Medical Center, Durham, NC USA; 2 Duke Global Health Institute, Box 3499, Durham, NC 27710 USA; 3 Center for Health Policy and Inequalities Research, Duke University, Durham, NC USA; 4 Center for Child and Family Health, Durham, NC USA; 5 Family Health International 360, Durham, NC USA; 6 Department of Biostatistics and Bioinformatics, Duke University, Durham, NC USA; 7 Department of Psychiatry, Kilimanjaro Christian Medical Centre, Moshi, Tanzania; 8 Department of Pediatrics, Kilimanjaro Christian Medical Centre, Moshi, Tanzania; 9 Kilimanjaro Christian Medical University College, Moshi, Tanzania; 10 Kilimanjaro Clinical Research Institute, Kilimanjaro Christian Medical Centre, Moshi, Tanzania

**Keywords:** Mental health, HIV, Adolescent, Africa, Tanzania

## Abstract

Sauti ya Vijana is a mental health and life skills intervention delivered by young adult group leaders for the improvement of HIV outcomes in young people living with HIV in Tanzania. This pilot randomized controlled trial estimated exploratory intervention effectiveness compared to standard of care. YPLWH (*N* = 105) were randomized to receive intervention or SOC. The mean age of participants was 18.1 years and 53% were female. Mean scores on mental health measures (*Patient Health Questionnaire* [PHQ-9], *Strengths and Difficulties Questionnaire* [SDQ], UCLA Trauma) were asymptomatic to mild in both study arms through 30-month follow-up with a non-significant fluctuation of 1–2 points. The mean self-reported adherence was higher in the intervention arm across all time points (but the confidence interval contained the null at all time points except 6 months). Risk ratio of virologic suppression (HIV RNA < 400 copies/mL) in the intervention arm compared to SOC was 1.15 [95% CI = 0.95, 1.39]) at 6-months, 1.17 [95% CI: 0.92, 1.48] at 12-months, and 0.99 [95% CI 0.76, 1.31] at 18-months. Though these findings were not powered for statistical significance, the trends in HIV outcomes suggest that SYV holds promise for improving antiretroviral therapy (ART) adherence and virologic suppression in YPLWH.

**Supplementary Information:**

The online version contains supplementary material available at 10.1007/s10461-021-03550-z.

## Introduction

An estimated 460,000 young people between 10–24 years of age became infected with HIV in 2019 [1]. These newly diagnosed young people, coupled with the growing number of perinatally HIV-infected children who have now reached 10–24 years of age, constitute the HIV-related “youth bulge.” Transitioning through the adolescent developmental period with HIV infection brings unique challenges surrounding HIV-related stigma, disclosure, and an increased risk of mental health difficulties, all of which affect both adherence to antiretroviral therapy (ART) and virologic suppression [2–4].

Mental health difficulties in the adolescent population remain largely unrecognized and untreated, especially among the estimated 5 million young people living with HIV (YPLWH) [5, 6]. Social determinants including poor social support, orphanhood, and poverty further exacerbate mental health difficulties [7, 8]. Unfortunately, few interventions address the mental health needs of YPLWH, and interventions are particularly sparse for those in low income settings. With an ongoing shortage of mental health professionals in low- and middle-income countries, non-specialist peers trained to deliver effective interventions are a critical implementation strategy for effective interventions [9].

Building on a prior paper summarizing 6-month outcomes [10], this current paper presents the exploratory, longitudinal outcomes of a randomized, controlled, pilot trial designed to evaluate the Sauti ya Vijana (SYV: The Voice of Youth) mental health and life skills intervention for YPLWH in Tanzania compared to standard of care (SOC) over a 30-month follow-up period.

## Methods

### Study Design and Setting

**Fig. 1:**
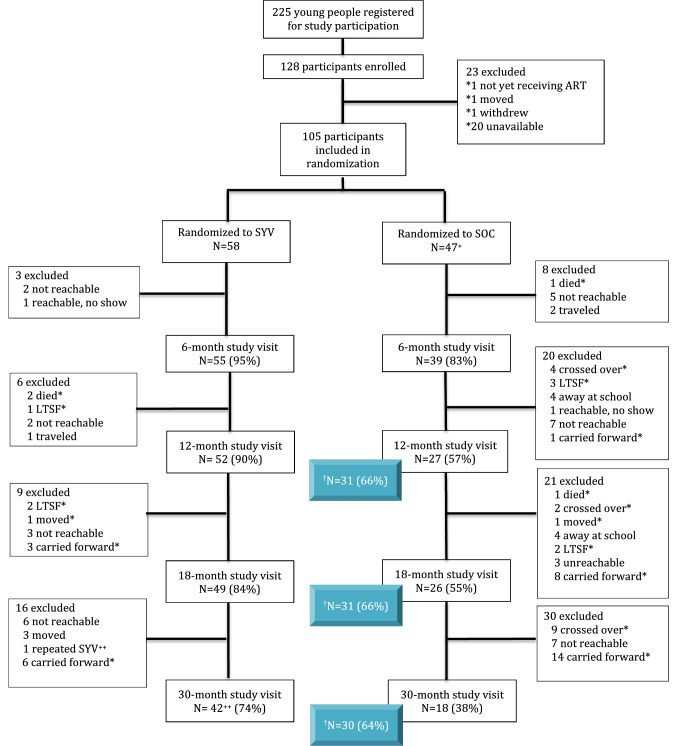
CONSORT flow diagram showing the number of participants excluded at each study visit compared to participants at randomization. ^+^1 participant missing baseline data, but study enrollment data available 6-months prior to intervention start. ^++^1 participant did not complete SYV intervention and joined a later cross-over wave. *did not contribute data at future time points (4 died (2 SOC & 2 SYV); 8 were lost to study follow up (LTSF); ^†^Shaded in blue: 15 crossed over during the timeframe of study follow-up. If data available from the cross-over group follow-up visits were included then the 12-month study follow-up would be 66%; 18-month study follow up would be 66%; 30-month follow up would be 64%

The study was an individually randomized, group treatment, pilot trial with three randomization waves spaced 6 months apart (Fig. 1 and Supplemental Fig. 1). Enrolled participants were randomized to the SYV intervention or standard of care (SOC) within one month prior to the start of their wave. All participants randomized to SOC were later offered the SYV intervention in a crossover wave at a time that enabled randomized comparisons between SYV and SOC (for details see Supplemental Fig. 1). Importantly, although the study used a stepped wedge design and collected data after crossover, the current paper excludes participant data obtained from the post-crossover periods of the SOC arm in order to compare the durability of findings in the intervention versus SOC arm over time.

The study took place in Moshi, Tanzania at two adolescent HIV clinics that offer a Saturday clinic solely for adolescents 12–24 years of age. The adolescent HIV clinic includes group health talks and peer support networks as part of a routine visit. Kilimanjaro Christian Medical Centre (KCMC) is the northern zone referral hospital serving approximately 430 YPLWH, and Mawenzi Regional Referral Hospital (MRRH) is the regional government hospital for the Kilimanjaro region serving approximately 320 YPLWH. Antiretroviral therapy (ART) is free in Tanzania, and adherence counselling accompanies all clinical visits as part of the Tanzanian national guidelines [11]. Currently, no mental health screening occurs in routine clinical care.

### Population

As previously described, YPLWH 12–24 years of age who were fully disclosed and aware of their HIV status and who attended the adolescent HIV clinic at KCMC or MRRH were approached to participate [10]. Recruitment occurred from May to July 2016 through the announcement of the study during the adolescent HIV clinic. Those interested joined a study enrollment list and were grouped by age categories (12–14 years; 15–19 years; 20–24 years of age), sex, and clinic site in the order in which they appeared on the list. All young people attending the clinic who were receiving ART and could understand and fully participate in the study intervention and informed consent process were eligible. Enrollment was not limited to those with identified mental health or adherence challenges because the intervention content was assumed to potentially benefit all YPLWH, not only those who self-reported challenges. The target enrollment was 100 participants (50 per arm) in order to obtain sufficient pilot data for the establishment of feasibility and acceptability of the intervention, and to explore immediate and prolonged outcomes. The data were intended to be used to judge whether to proceed with a future definitive trial. Enrollment and randomization were increased to 130 during the study due to attrition.

### Procedures

Five evaluation visits occurred at: (1) baseline (pre-intervention), (2) 6 months, (3) 12 months, (4) 18 months, and (5) 30 months post-baseline, with baseline separated by 6 months between waves. Intervention was completed prior to the 6 month visit in all waves (Fig. 1 and Supplemental Fig. 1). Participants responded to a structured interview conducted by one of two research assistants in Kiswahili, the official language of Tanzania. The interview included questions about demographics, risk behaviors, perceptions of internal and external stigma, mental health symptoms, and ART adherence. A 5 mL blood sample was drawn to measure HIV RNA. The two research assistants conducting the study evaluation visits were blinded to the participants’ study arm assignment.

### Randomization and Masking

Using the study enrollment list, participants were organized into one of three waves of males and females of similar age (Supplemental Fig. 1 for details) at each of the two sites (KCMC or MRRH). Then, within each wave and site, the males and females were separately randomized to SYV or standard of care (SOC) by coin flip just prior to the start of each wave in the presence of the principal investigator and group leaders. For the male MRRH group (Wave 2), only nine of the participants were reachable and able to participate at the time of randomization, thus there is no control group (Supplemental Fig. 1). Participants randomized to SYV received the intervention in a group of 9–11 participants, and participants randomized to SOC continued with usual attendance of the monthly adolescent HIV clinic. Participants in the SOC arm were later offered to participate in the SYV intervention as a cross-over wave (Supplemental Fig. 1). There was no time and attention-matched control group, meaning that the SOC arm did not meet as a study group.

### Intervention

The SYV intervention is a group intervention involving ten group sessions, two designed to be held jointly with caregivers, two individual sessions between the participant and a group leader, and a final celebration to summarize intervention content and distribute completion certificates [12]. Sessions were designed based on formative research and youth advisor input [3, 13]. Components of evidence-based models—including Cognitive Behavioral Therapy, Interpersonal Psychotherapy, and Motivational Interviewing—were chosen based on the best fit for specific youth-identified needs and on whether they could be implemented with fidelity by lay group leaders [14]. A description of the manualized session-by-session intervention protocol has been previously described [10, 15], and the full intervention protocol is available upon request to the corresponding author. Sessions lasted approximately 90 min and were held on consecutive Saturdays for a duration of 10 weeks at a non-clinical site (see Supplemental Fig. 1 for detailed dates). This resulted in approximately 18.5 contact hours in the intervention group. Fidelity to the intervention was marked on a fidelity checklist by the observing group leader. The six month study visit occurred after completion of the intervention in all waves. Enrolled young people who missed more than two consecutive intervention sessions were dropped from their assigned group to preserve group rapport and forward momentum, as the intervention content builds upon prior sessions. Young people who prematurely ended SYV were offered the possibility of joining a later cross-over control group.

The study was implemented with Tanzanian group leaders who were trained to deliver the intervention and were closely supervised twice weekly. Six young adults (age 23 to 30 years) with a background of either living with HIV and/or having prior experience with mental health research were trained during an intensive two-week training on-site with the principal investigator and the US-based clinical psychologist. Two group leaders led the group-based intervention session, and one group leader marked the fidelity checklist and took session notes at each session. The fidelity checklist and session notes were used to guide the weekly supervision meetings that occurred both in person with the group leaders and principal investigator, and using telecommunication via Skype to communicate with the US-based clinical psychologist.

### Measures

Demographic measures including primary caregiver, social support (if you are feeling sad, is there someone you can talk to?), home environment (availability of plumbing and electricity), livelihood (whether in school or working), cell phone ownership, sexual history, and alcohol and drug use were documented at every study visit. No changes were made to the study measures over the course of the trial.

Mental health measures were chosen based on published research and harmonization with other adolescent mental health projects in Africa. Justification for use in this trial has been presented in more detail previously [3, 10]. The *Patient Health Questionnaire* (PHQ-9) was used to measure symptoms of depression. There has been published research on use in adolescent populations in Kenya, Ghana, Zimbabwe, and the Republic of Congo [4, 16–18], in addition to our prior research in Tanzania [3]. This measure includes nine questions with a response range of 0–27. A score of 10 or greater is commonly used as a screening threshold suggestive of moderate to severe depressive symptoms [19, 20]. The *Strengths and Difficulties Questionnaire* (SDQ) was used to measure emotional and behavioral symptoms. The scale includes 25 questions (including 5 prosocial behavior questions that are not included in the composite score). A cut-off of 17 or higher (score range of 0–40) signals symptomatology of mental health difficulties with mean Cronbach α of 0.73 as assessed by the tool developers [21], signifying the internal consistency of this tool. Trauma related symptoms were assessed using the *UCLA Post Traumatic Stress Symptoms Exposure Screener and Reaction Index,* and survey responses were modified to a four-question Likert scale (None, Some, Much, and Most) based on translation from Swahili [22, 23]. A cut-off of 18 or greater was suggestive of post-traumatic stress related symptoms [3]. Prior use of the tool internationally demonstrated inter-rater reliability and criterion-related validity with children in Zambia [24], and internal consistency was demonstrated with its use among adolescent Somalian refugees (Cronbach α = 0.85) [25].

Stigma was measured using 10 questions from the *Berger HIV Stigma Scale*. Questions related to internal stigma or “negative self-image” (four questions) and external stigma or “public attitudes” (six questions) were analyzed in these two categories and as a total stigma score (scale range 10–40) [3, 10, 26].

ART adherence was measured by a validated three-question survey that asked participants to rate their adherence over the past 30 days, translated to a 0–100 scale [27]. The mean of these three questions was the value used to measure self-reported adherence, with a higher number indicating better adherence. HIV RNA measurement was performed at the Kilimanjaro Clinical Research Institute Biotechnology Laboratory, which participates in international external quality assurance programs and uses the Abbott m2000 platform (Des Plaines, Illinois, USA). Virologic suppression was defined as plasma HIV RNA level < 400 copies/ml. HIV RNA lower than the minimal detectable amount of 40 copies/ml was imputed as half the minimum detectable amount (20 copies/ml).

### Statistical Analysis

We summarized continuous variables using means, standard deviations, medians, and quartiles, and categorical variables using counts and percentages. Data were analyzed as intention-to-treat according to the participant’s randomization assignment, regardless of whether the participant attended or participated in the intervention if assigned to the intervention arm. Data from all participants contributing data at each timepoint were utilized, except after the time of cross-over for participants randomized to SOC who were later exposed to the intervention (Fig. 1).

A constrained longitudinal linear mixed effects model with clustering in the intervention arm was used for continuous outcomes [28]. This model takes into account clustering using a random intercept for the group in which participants in the SYV-arm received the group-based intervention, while the individual-level variability is allowed to vary between the SYV intervention and SOC arms [29]. The longitudinal nature of the data was accounted for using non-independent structure on the residuals within person. Since many of the continuous outcomes are skewed, we used robust standard errors and checked residuals. All models were adjusted for wave and site.

The binary variable of virologic suppression was analyzed using the modified Poisson approach, namely a log-Poisson generalized estimating equations (GEE) model with robust standard errors [30]. The log-link is used instead of the logit because of the well-known issue of misinterpretation of the odds ratio [31]. The model took into account correlation within participants over time using unstructured covariance, and was also adjusted for wave and site to account for these design characteristics.

We examined pre-intervention predictors of missingness at each time point and as a sensitivity analysis adjusted for variables in the statistical model with a p-value of the difference between missing and non-missing of < 0.10 at any time point. As additional sensitivity analyses, the intervention effect was examined including those who crossed-over as remaining in the control arm. One participant in Wave 2 did not have baseline data collected, but did have study entry data six-months prior. These data are included in the summary table, but study entry data are not included in the regression models.

Due to the pilot nature of the study, all analyses are exploratory. Analyses were conducted using Stata version 16.1 (StataCorp, College Station, TX).

## Results

A total of 105 participants were randomized to SYV intervention or SOC. At baseline, the average age of participants was 18.1 years (standard deviation [SD] = 2.3 years; range 13–24 years), and approximately half (53%) of the participants were female with the majority perinatally HIV infected (approximately 86%). The age of participants in each group can be found in Supplemental Fig. 1. Three groups included 13- and 14-year-old participants with older adolescents of 18 to 19 years of age. Over time, fewer females contributed data, and eight females had babies during the course of the study. Cell phone ownership became more common over time; 93% at the 30-month follow-up compared to 66% at baseline (Table 1). As the cohort aged, more youth reported having engaged in sexual intercourse (57% at 30-months compared to 29% at baseline), having used a condom during the last sexual encounter (85% at 30 months compared to 70% at baseline) and alcohol use (23% at 30 months versus 11% at baseline). Summaries of behavioral, mental health, stigma, and adherence outcomes at each time point by intervention arm are provided in Supplemental Tables Ia–Ie.

**Table 1:** Demographics of participants contributing data at each study time point

	Baseline	6 months	12 months	18 months	30 months
	*N* = 105	*N* = 94	*N* = 79	*N* = 75	*N* = 60
Age^a^ (years)					
Mean (standard deviation)	18.1 (2.3)	18.4 (2.2)	19.0 (2.2)	19.6 (2.4)	20.6 (2.5)
Median (Quartile 1, Quartile 3)	17.7 (16.5, 19.3)	18.1 (16.8, 19.6)	18.4 (17.3, 20.3)	19.3 (17.9, 21.2)	20.1 (18.9, 21.7)
Gender (female)	56 (53%)	48 (51%)	40 (51%)	37 (49%)	27 (45%)
Primary caregiver (biological parent)	51 (49%)	48 (51%)	40 (51%)	41 (55%)	24 (40%)
Social support (have someone to talk with)	85 (81%)	77 (82%)	66 (84%)	66 (88%)	52 (87%)
Perinatally HIV infected	90 (86%)	84 (89%)	73 (92%)	70 (93%)	55 (92%)
Home environment (no plumbing nor electricity)	18 (17%)	17 (18%)	9 (11%)	9 (12%)	5 (8%)
Neither in school nor working	25 (24%)	25 (27%)	23 (29%)	28 (37%)	18 (30%)
Cell phone ownership	69 (66%)	63 (67%)	59 (75%)	61 (81%)	56 (93%)
Ever had sexual intercourse	30 (29%)	30 (32%)	22 (28%)	27 (36%)	34 (57%)
Condom used during most recent sexual encounter^b^	21 (70%)	21 (70%)	17 (77%)	19 (70%)	29 (85%)
Alcohol use (last 6 months)	11 (11%)	7 (7%)	7 (9%)	10 (13%)	14 (23%)
Drug use (last 6 months)	0 (0%)	1 (1%)	2 (3%)	0 (0%)	2 (3%)

Measures all reported as count (percent) unless otherwise specified; patients may contribute data intermittently

^a^Mean (standard deviation)

^b^Denominator from those reporting sexual activity

### Feasibility, Acceptability, and Descriptive Outcomes of Longitudinal Study Follow-Up

Among those randomized to receive the intervention, four participants never attended a session (3 in Wave 1; 1 in Wave 2) and three participants were dropped due to missing more than two consecutive sessions (2 participants missed session 7–9 and were dropped; 1 participant missed sessions 2–4 and was dropped). Nearly 90% of YPLWH randomized to receive intervention received completion certificates (51/58) with an average of 95% attendance for all sessions. The 6-month study visit was attended by 90% of participants in the SYV arm compared to 83% in the SOC arm; 74% and 65% attended the 30-month study visit in the SYV and SOC arms respectively. Those randomized to SOC did not meet in a group and were more likely to miss study follow up visits due to being unreachable (Fig. 1). Several youth could not attend study follow-up because they were away at school, travelled, or moved. Eight participants (three randomized to intervention and five randomized to control) were lost to study follow-up (LTSF), meaning the study team lost contact, and these youth never returned to a future study visit (Fig. 1). There was no difference in HIV RNA at baseline between these participants and those who remained engaged in the study. The majority who were LTSF (7 of 8) were from the MRRH site. Based on the clinical files, three participants remained in standard clinical care at MRRH, but had no reachable phone; the other five participants were documented as having transferred to a distant clinic. Four participants (3.8%), two in each study arm, died during the study, all due to AIDS-related causes.

### Exploratory Effectiveness Outcomes

**Fig. 2:**
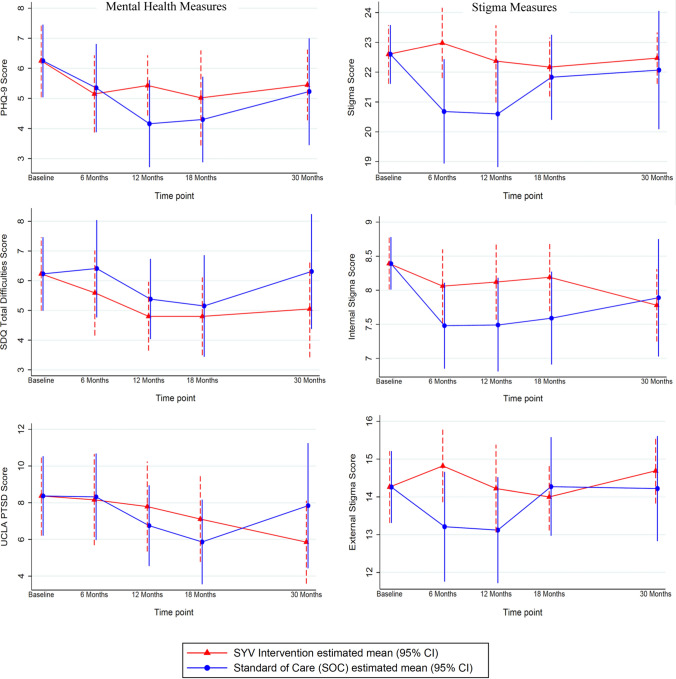
Mixed effects regression intervention effect estimates on mental health and stigma adjusted for wave and site

Adjusted mean mental health scores on all three measurement tools were in a range of asymptomatic to mild across time points in both arms (Supplemental Tables Ia–Ie and Fig. 2). The PHQ9 remained below that of baseline in both groups at all study time points, but was between 5 and 6 in the SYV arm compared to 4 to 5 in the SOC arm. Similar trends were seen regarding symptoms of post-traumatic stress (Fig. 2). Stigma scores remained consistent over time with non-significant 1–2 point fluctuations over time. Internal stigma down-trended from baseline in both arms at all study time points. Mixed effects regression of total stigma demonstrated a mean difference of 1.77 (95% CI = 0.01, 3.54) at 12 months, 0.35 (95% CI = −0.96, 1.66) at 18 months, and 0.4 (95% CI = −1.61, 2.41) at 30 months in the SYV arm compared to SOC; results that favor the SOC arm (Table 2, Fig. 2, Supplemental Tables Ia-Ie).

**Table 2:** Mixed effects regression of mental health, stigma, and adherence measures of the SYV intervention effect estimates compared to SOC^a^, adjusted for wave and site

Study measure		Intervention effect estimate^b^ at each timepoint (95% CI)			
		6 months	12 months	18 months	30 months
Mental health^a^	PHQ-9	− 0.19 (− 1.53, 1.15)	1.27 (0.04, 2.49)	0.71 (− 0.64, 2.07)	0.22 (− 1.48, 1.92)
	SDQ	− 0.82 (− 2.18, 0.55)	− 0.58 (− 1.77, 0.61)	− 0.35 (− 1.84, 1.15)	− 1.26 (− 3.38, 0.86)
	UCLA PTSD	− 0.16 (− 2.57, 2.25)	1.04 (− 1.52, 3.59)	1.25 (− 1.21, 3.71)	− 1.99 (− 5.68, 1.69)
Stigma	Total score	2.30 (0.66, 3.94)	1.77 (0.01, 3.54)	0.35 (− 0.96, 1.66)	0.40 (− 1.61, 2.41)
	Internal	0.58 (− 0.16, 1.32)	0.62 (− 0.10, 1.35)	0.60 (− 0.19, 1.38)	− 0.11 (− 1.07, 0.85)
	External	1.61 (0.40, 2.82)	1.10 (− 0.26, 2.46)	− 0.28 (− 1.37, 0.82)	0.47 (− 0.98, 1.92)
Adherence	Self-Report^a^	6.66 (1.40, 11.91)	3.54 (− 1.74, 8.82)	2.51 (− 2.93, 7.94)	3.22 (− 3.76, 10.19)
	HIV RNA^c^	− 0.86 (− 1.50, − 0.22)	− 0.12 (− 0.99, 0.76)	− 0.11 (− 1.14, 0.91)	N/A

^a^A negative value favours intervention compared to SOC except with respect to self-reported adherence for which a positive value favours intervention

^b^Estimated mean difference between SYC and SOC arms

^c^Log transformed

**Fig. 3:**
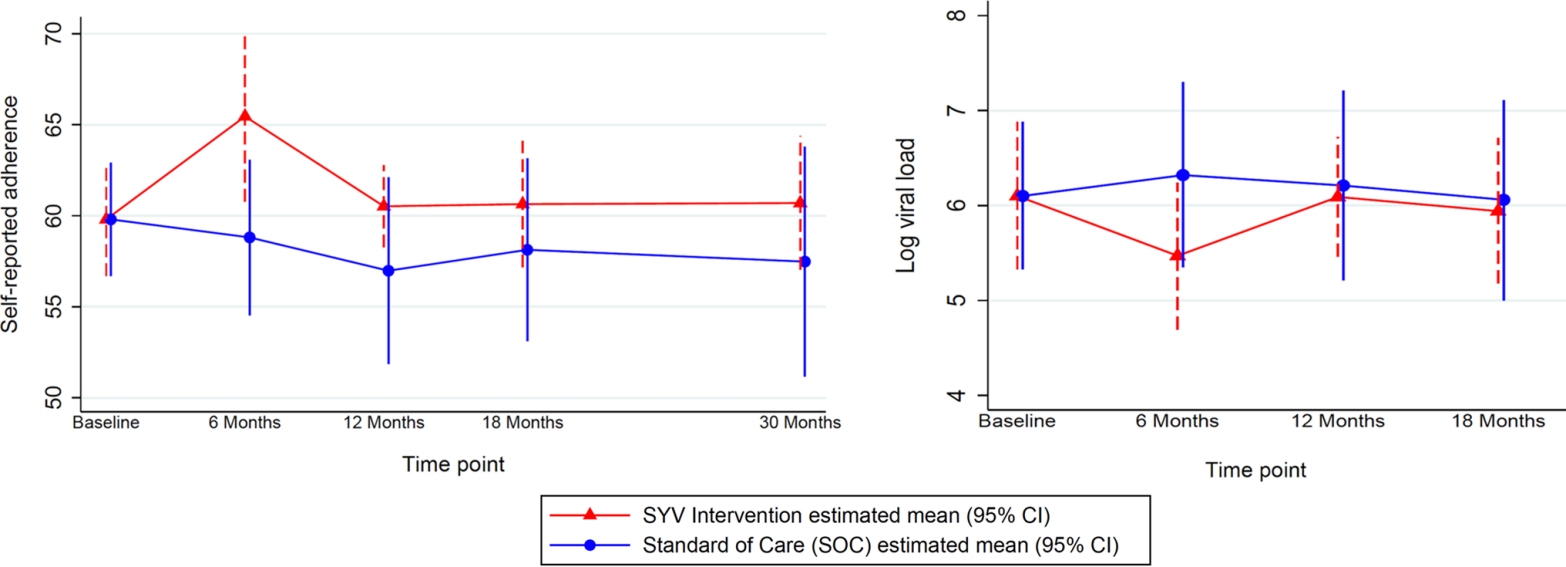
Mixed effects regression intervention effect estimates on adherence measures, adjusted for wave and site. An increase in self-reported adherence favors the intervention; a reduction in Log viral load (HIV RNA) favors the intervention

There was a self-reported adherence mean difference of 6.66 (95% CI = 1.40, 11.91) at 6 months in the intervention arm compared to SOC (Table 2, Fig. 3), and self-reported adherence trended higher in the intervention arm across all additional time points, although the confidence intervals at later time points contain the null (e.g., at 30 months, mean difference = 3.22 [95% CI = − 3.76, 10.19]).

Additionally, there was a mean difference in log transformed HIV RNA of −0.86 (95% CI = −1.50, −0.22) at 6 months and −0.11 (CI = −1.14, 0.91) at 18 months between the intervention arm compared to the SOC arm (Table 2, Fig. 3). Likewise, virologic suppression (HIV RNA < 400 copies/mL) was favored in the intervention arm at 6 months (risk ratio = 1.15 [95% CI = 0.95, 1.39]), 12 months (risk ratio = 1.17 [95% CI = 0.92, 1.48]), and at 18 months (risk ratio = 0.99 [95% CI 0.76, 1.31]) with wide confidence intervals as expected due to the pilot nature of the trial. No major harms were found during the pilot trial; however, an unintended effect of the intervention may have been an increased awareness of external stigma in the SYV arm as compared to SOC. Three baseline variables were found to be differential by missingness at any time point (cell phone ownership, gender, and alcohol use). Results did not change appreciably in sensitivity analysis.

## Discussion

In this pilot trial, exploratory effectiveness analyses point to the potential of the SYV intervention to improve adherence and virologic suppression. Self-reported adherence trended higher in the intervention arm across all time points, although the confidence interval contains the null at all time points except 6 months. The trends in HIV RNA favored virologic suppression in the intervention arm compared to the control arm, but importantly, the study was underpowered to indicate statistical significance.

The feasibility and acceptability of the intervention and study design are supported by the study findings. Ninety percent of YPLWH randomized to intervention attended an average of 95% of the sessions that spanned approximately 3 months. There was approximately 5% attrition at each 6-month follow-up interval in the intervention arm, but those randomized to the intervention arm were more likely to attend study follow-up visits compared to those randomized to SOC. This may be because YPLWH in the intervention arm were more engaged in the study, with more contact hours from attending group sessions versus study visits alone.

The study incorporated a broad age range (13–24 years) that reflected the age of YPLWH attending the adolescent HIV clinics from which the study recruited. Some intervention groups included younger adolescents (13- and 14-year-olds) with older adolescents (18- and 19-year-olds). Despite this broad chronological age range, the developmental age within groups was well aligned and the diversity of life experiences discussed in the groups was helpful for the entire group. Chronologic age does not always match developmental age, and younger participants may have more life experience (for example sexual experiences, romantic relationships, experience with disclosure) than older participants in the group.

Few longitudinal studies exist in this highly mobile and dynamic population. This study suggests longitudinal engagement is possible, but additional strategies could improve retention. Some youth who were not reachable had moved to another city for work or school. but for many, the original contact information was no longer accurate. Maintaining monthly communication is critical to ensure up to date contact information of the participant, and we learned that having contact information for at least two or three trusted people for each participant is important to facilitate longitudinal follow-up and improve study retention. By the end of the study, almost all participants contributing data reported owning a cell phone (93%). Utilizing lessons learned during the COVID-19 pandemic, phone-based study assessments might be possible for those who are away at school or traveling [32].

Enrollment criteria were not limited to those who reported mental health symptoms at baseline. As mental health symptoms are common in adolescents, with up to 50% of all mental health conditions occurring before the age of 14 years [12, 33], we hypothesized that SYV could benefit all YPLWH by raising awareness about mental health and teaching coping strategies that could prevent or dampen symptoms that might arise in those asymptomatic at baseline. The mean scores on all three mental health measures were in a range considered to be asymptomatic to mild in this study. Fluctuations of one or two points occurred in the mental health measures, but were not considered to represent clinically meaningful change and were not statistically significant.

Similar to mental health measures, stigma reported by participants remained fairly consistent over time, fluctuating by one or two points. Many youth were not previously aware of their internal stigma, or negative self-talk, but they came to recognize it during the intervention. Youth identified education as a way to reduce external stigma in the community. However, it was evident that fear of inadvertent disclosure (people assuming they were HIV-infected) if they were to educate their community about HIV and the consequences for their social relationships was a significant barrier. Understanding this integral connection between stigma and disclosure, and external stigma being a driver of internal stigma, are important areas for further exploration.

The pilot trial had several limitations. Not all participants were available to cross-over, and thus the trial was not analyzed as a pure stepped-wedge design. Exclusion of data from the cross-over group meant that data from the SOC arm was reduced over time. Due to budget limitations, the 30-month HIV RNA sample was not analyzed. Many of the study findings rely on self-reporting during the structured interview and were subject to reporter bias. Outcome measures in the pilot trial did not explicitly measure coping or resilience, and these will be important factors to consider as mediating pathways when planning an appropriately powered effectiveness trial (Supplemental Fig. 2).

Despite the benefits of SYV, 3.8% of the participants died over the 30 months of follow-up. The latest statistics from UNAIDS suggests that a young person dies every 10 min due to an AIDS-related illness [34]. This is an unacceptable reality at a time when we have the necessary tools for HIV to be a treatable, chronic illness with a life span that mirrors that of HIV negative peers. We must do better to identify those YPLWH who are not adherent to ART and intervene before viral resistance or severe symptoms begin. The situation comes with the mandate to work harder to identify, address, and prevent mental health challenges, and to empower YPLWH with coping strategies and skills that enable them to choose a healthy and hopeful life.

## Conclusions

SYV holds promise towards improving HIV-related outcomes of adherence and virologic suppression. Intervention effects tended to dampen by 12 to 18 months, so more frequent communication and periodic intervention boosters may help keep youth engaged and able to experience prolonged benefit. Such amendments will be incorporated into a future effectiveness trial of the SYV intervention.

## Supplementary Information

Below is the link to the electronic supplementary material.

Supplementary file1 (PDF 351 kb)

## Data Availability

Deidentified participant data collected for the study, the data dictionary, and the statistical analysis plan will be made available through UNC Dataverse in the AMANI collection (https://dataverse.unc.edu/dataverse/amani) expect to be available at the time of publication. Additional data is available by emailing the corresponding author at Dorothy.dow@duke.edu. This dataset will be available in the open-source AMANI Collection on Dataverse (https://dataverse.unc.edu/dataverse/amani).

## Code Availability

It will be available in the open-source AMANI Collection on Dataverse (https://dataverse.unc.edu/dataverse/amani).
