# Supplementary material for: Sauti ya Vijana (SYV; The Voice of Youth): Longitudinal Outcomes of an Individually Randomized Group Treatment Pilot Trial for Young People Living with HIV in Tanzania
Source: AIDS Behav. 2022 Jan 24;26(6):2015–25. doi: 10.1007/s10461-021-03550-z (PMC8784208; doi:10.1007/s10461-021-03550-z)
Supplement: Supplementary file 1 — Supplementary file1 (PDF 351 kb) [file 10461_2021_3550_MOESM1_ESM.pdf]

Supplemental Table Ia. Summaries of outcomes and variables of interest by intervention arm **at baseline**

	Control (N = 47)	Intervention (N = 58)	Total (N = 105)
**PHQ-9**	6.6 (4.3)	5.1 (3.5)	5.8 (3.9)
**SDQ**	7.3 (3.7)	7.2 (3.9)	7.3 (3.8)
**UCLA PTSD**	9.9 (7.5)	8.5 (7.3)	9.2 (7.4)
**Total Stigma Score**	23.4 (4.5)	21.9 (4.9)	22.6 (4.8)
**Internal Stigma Score**	8.2 (2.0)	7.8 (1.9)	8.0 (1.9)
**External Stigma Score**	15.2 (4.0)	14.3 (4.2)	14.7 (4.1)
**Self-reported adherence**	58.3 (15.4)	60.2 (11.4)	59.3 (13.3)
**HIV RNA copies/mL (log transformed)**	4.9 (2.8)	5.4 (3.3)	5.2 (3.1)
**HIV RNA < 400 copies/mL**	33 (70.2%)	37 (64.9%)	70 (67.3%)
**Ever had sexual intercourse**	17 (36.2%)	13 (22.8%)	30 (28.8%)
***Condom used during most recent sexual encounter**	11 (64.7%)	10 (76.9%)	21 (70.0%)
**Social support (someone to go to for help)**	39 (83.0%)	46 (79.3%)	85 (81.0%)

Note: Measures are reported as means and standard deviations, except where indicated by a % in the parentheses, which are reported as N (%). *Denominator from those reporting sexual activity.

Supplemental Table Ib. Summaries of outcomes and variables of interest by intervention arm **at 6 months**

	Control (N = 39)	Intervention (N = 55)	Total (N = 94)
**PHQ-9**	5.2 (4.0)	4.1 (3.4)	4.6 (3.7)
**SDQ**	7.3 (4.3)	6.7 (4.4)	7.0 (4.4)
**UCLA PTSD**	9.0 (6.2)	8.6 (7.5)	8.7 (7.0)
**Total Stigma Score**	21.6 (5.1)	22.7 (5.3)	22.2 (5.2)
**Internal Stigma Score**	7.1 (2.0)	7.5 (2.1)	7.4 (2.1)
**External Stigma Score**	14.4 (4.2)	15.1 (4.5)	14.8 (4.4)
**Self-reported adherence**	57.8 (14.7)	65.5 (12.3)	62.3 (13.8)
**HIV RNA copies/mL (log transformed)**	5.4 (3.3)	4.7 (2.6)	5.0 (2.9)
**HIV RNA < 400 copies/mL**	25 (64.1%)	41 (74.5%)	66 (70.2%)
**Ever had sexual intercourse**	16 (41.0%)	14 (25.9%)	30 (32.3%)
***Condom used during most recent sexual encounter**	10 (62.5%)	11 (78.5%)	21 (70.0%)
**Social support (someone to go to for help)**	31 (79.5%)	46 (83.6%)	77 (81.9%)

Note: Measures are reported as means and standard deviations, except where indicated by a % in the parentheses, which are reported as N (%). *Denominator from those reporting sexual activity.

Supplemental Table Ic. Summaries of outcomes and variables of interest by intervention arm **at 12 months**

	Control (N = 27)	Intervention (N = 52)	Total (N = 79)
**PHQ-9**	3.5 (2.8)	4.6 (3.5)	4.2 (3.3)
**SDQ**	5.7 (3.6)	5.9 (3.5)	5.8 (3.5)
**UCLA PTSD**	6.5 (4.6)	8.0 (7.6)	7.5 (6.7)
**Total Stigma Score**	21.2 (4.9)	22.1 (4.9)	21.8 (4.9)
**Internal Stigma Score**	7.1 (1.6)	7.7 (1.8)	7.5 (1.7)
**External Stigma Score**	14.1 (4.3)	14.4 (4.5)	14.3 (4.4)
**Self-reported adherence**	57.3 (15.8)	60.3 (14.2)	59.2 (14.7)
**HIV RNA copies/mL (log transformed)**	5.4 (3.1)	5.0 (3.1)	5.1 (3.1)
**HIV RNA < 400 copies/mL**	16 (59.3%)	39 (75.0%)	55 (69.6%)
**Ever had sexual intercourse**	11 (40.7%)	11 (21.2%)	22 (27.8%)
***Condom used during most recent sexual encounter**	8 (72.7%)	9 (81.8%)	17 (77.3%)
**Social support (someone to go to for help)**	24 (88.9%)	42 (80.8%)	66 (83.5%)

Note: Measures are reported as means and standard deviations, except where indicated by a % in the parentheses, which are reported as N (%). *Denominator from those reporting sexual activity.

Supplemental Table Id. Summaries of outcomes and variables of interest by intervention arm **at 18 months**

	Control (N = 26)	Intervention (N = 49)	Total (N = 75)
**PHQ-9**	4.0 (2.4)	4.3 (3.5)	4.2 (3.1)
**SDQ**	6.3 (4.5)	5.9 (4.2)	6.0 (4.3)
**UCLA PTSD**	7.0 (6.6)	7.3 (7.0)	7.2 (6.8)
**Total Stigma Score**	22.3 (4.2)	22.3 (4.9)	22.3 (4.6)
**Internal Stigma Score**	7.2 (1.6)	7.8 (1.7)	7.6 (1.7)
**External Stigma Score**	15.3 (4.1)	14.5 (4.0)	14.8 (4.0)
**Self-reported adherence**	57.8 (13.5)	60.7 (10.6)	59.7 (11.7)
**HIV RNA copies/mL (log transformed)**	4.9 (2.9)	5.2 (3.3)	5.1 (3.1)
**HIV RNA < 400 copies/mL**	17 (68.0%)	32 (68.1%)	49 (68.1%)
**Ever had sexual intercourse**	11 (42.3%)	16 (32.7%)	27 (36.0%)
***Condom used during most recent sexual encounter**	6 (54.5%)	13 (81.3%)	19 (70.4%)
**Social support (someone to go to for help)**	23 (88.5%)	43 (87.8%)	66 (88.0%)

Note: Measures are reported as means and standard deviations, except where indicated by a % in the parentheses, which are reported as N (%). *Denominator from those reporting sexual activity.

Supplemental Table Ie. Summaries of outcomes and variables of interest by intervention arm **at 30 months**

	Control (N = 18)	Intervention (N = 42)	Total (N = 60)
**PHQ-9**	5.4 (4.5)	4.7 (3.9)	4.9 (4.1)
**SDQ**	7.2 (4.9)	6.0 (4.0)	6.4 (4.3)
**UCLA PTSD**	7.7 (8.2)	6.0 (6.5)	6.5 (7.0)
**Total Stigma Score**	21.8 (5.4)	22.3 (4.7)	22.2 (4.9)
**Internal Stigma Score**	7.6 (1.9)	7.4 (1.8)	7.5 (1.9)
**External Stigma Score**	14.4 (4.4)	14.9 (4.4)	14.8 (4.3)
**Self-reported adherence**	57.2 (14.0)	60.5 (13.2)	59.5 (13.4)
**HIV RNA copies/mL (log transformed)**	N/A	N/A	N/A
**HIV RNA < 400 copies/mL**	N/A	N/A	N/A
**Ever had sexual intercourse**	13 (72.2%)	21 (50.0%)	34 (56.7%)
***Condom used during most recent sexual encounter**	10 (76.9%)	19 (90.5%)	29 (85.2%)
**Social support (someone to go to for help)**	15 (83.3%)	37 (88.1%)	52 (86.7%)

Note: Measures are reported as means and standard deviations, except where indicated by a % in the parentheses, which are reported as N (%). *Denominator from those reporting sexual activity.

Months		July-Aug	Sep-Dec	Jan	Feb-May	Jun-July	Aug-Nov	Dec-Jan	Feb-Apr	May-July	Dec-Jan	May-Aug	Sept	Dec-Jan	Jan-April
Year		2016		2017					2018			2019			2020
**Wave 1**		**Entry/ Baseline**		**6-mo**		**12-mo**		**18-mo**			**30-mo**				
	**Male SYV** [+]Mean age 20 (18 - 24 years)	x	SYV Int	x		x		x			x				
	**Female SYV** [+]Mean age 16 (14 -18 years)	x		x		x		x			x				
	Male SOC	x		x		x		x	SYV Int*	x*	x*	SYV Int**	x**		SYV Int***
	Female SOC	x		x		x		x		x			x**		
**Wave 2**		**Entry**		**Baseline**		**6-mo**		**12-mo**		**18-mo**		**30-mo**			
	**Male SYV** [+]Mean age 17 (13 - 18 years)	x		x	SYV Int	x		x		x		x			
	**Female SYV** [+]Mean age 17 (16 - 18 years)	x		x		x		x		x		x			
	Female SOC	x		x		x		x	SYV Int*	x*	x*	SYV Int**	x**		SYV Int***
**Wave 3**		**Entry**				**Baseline**		**6-mo**		**12-mo**	**18-mo**			**30-mo**	
	**Male SYV** [+]Mean age 17 (14 - 19 years)	x				x	SYV Int	x		x	x			x	
	**Female SYV** [+]Mean age 18 (16 - 19 years)	x				x		x		x	x			x	
	Male SOC	x				x		x	SYV Int*	x*	x*	SYV Int**	x**		SYV Int***
	Female SOC	x				x		x		x	x		x**		

Supplemental Figure 1: Sauti ya Vijana, Stepped-Wedge Study Design with Average and Age Range Represented in Each Group

[+]Mean age and in parenthesis (age range) in years rounded to the whole number. The SOC mean age and range is reflected in the SYV intervention group

Abbreviations: SYV = Sauti ya Vijana intervention; SOC = standard of care “control” group; mo = month. Baseline includes 88 participants; an additional 40 participants enrolled over time to complete groups for waves 2 and 3.

Shading: Wave 1 in pink, Wave 2 in orange (note, no male SOC group), Wave 3 in green with study follow visits shaded for those who received intervention and remaining clear for the control group; partial shading signifies one or more participants, but not all crossed over. *First crossover wave (Wave 4) in blue (3 males from W1; 4 males from W3; 2 females W2 all from KCMC site); **Second crossover wave (Wave 5) in purple (run as a mixed gender and mixed site group); ***A third and final cross over wave (Wave 6) (run as a mixed gender and mixed site group) with a pause due to COVID-19 safety restrictions.

Study questionnaire and outcome measures at entry/baseline, 6-, 12-, 18-, and 30-mo follow up (+/- 6 weeks due to school exams and holidays) are in corresponding color blocks to indicate data in the present manuscript; cross over data are currently being analyzed.

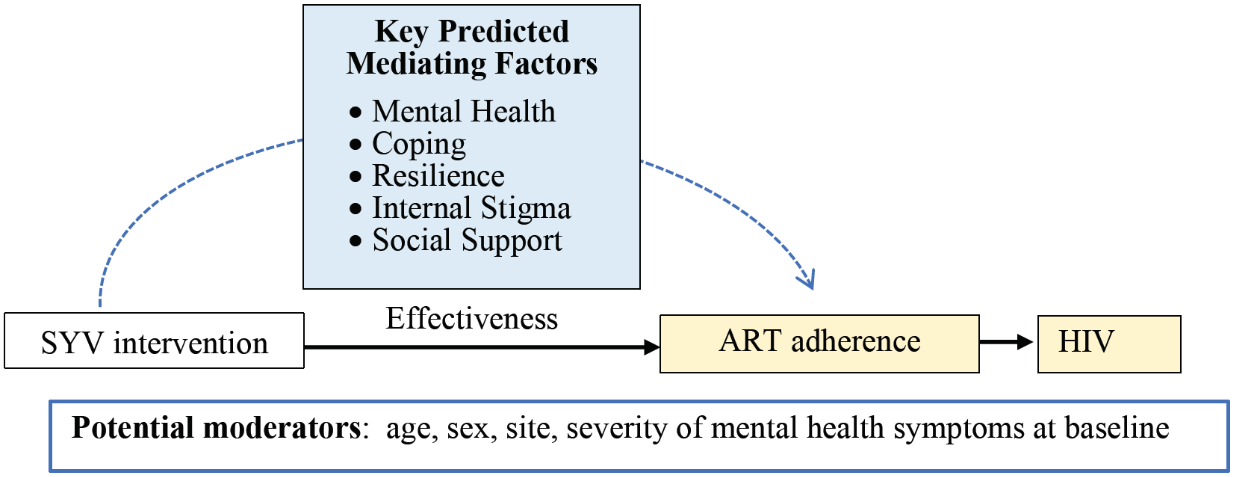

Supplemental Figure 2: SYV Intervention Proposed Mechanism of Change
